# Value of Central Venous Pressure Monitoring in the Patients with Sepsis-Associated Acute Kidney Injury

**DOI:** 10.1155/2022/9652529

**Published:** 2022-12-10

**Authors:** Yaya Xu, Xiangmei Kong, Xiaodong Zhu, Jianhua Zhang, Yueniu Zhu

**Affiliations:** ^1^Department of Pediatric Critical Care Medicine, Xinhua Hospital Affiliated to the Medical School of Shanghai Jiaotong University, 1665 Kongjiang Road, Yangpu District, Shanghai 200092, China; ^2^Department of Pediatric Respiratory Department, Xinhua Hospital Affiliated to the Medical School of Shanghai Jiaotong University, 1665 Kongjiang Road, Yangpu District, Shanghai 200092, China

## Abstract

**Background:**

Although the measurement of central venous pressure (CVP) is a common clinical tool, the role of CVP monitoring in the outcome of sepsis is controversial because threshold values of CVP are uncertain, and there are only limited data on short-term survival of patients with septic acute kidney injury (AKI).

**Methods:**

This retrospective cohort study was based on the Medical Information Mart for Intensive Care IV (MIMIC-IV) database (source of the training dataset). Multivariate regression analysis was performed to clarify the relation between CVP measurement and clinical outcomes, and a univariate regression model after propensity score matching was utilized to validate our findings. A mortality prediction model for septic AKI and a risk stratification scoring approach were developed, and the emergency intensive care unit (eICU) database was used for external validation.

**Results:**

Of the 9170 patients in the training set, 2446 (26.7%) underwent CVP measurement. No significant association was found between CVP monitoring and 28-day mortality among patients with septic AKI (odds ratio = 0.479; 95% confidence interval 0.213-1.076, *P* = 0.075), even after adjustments (propensity score matching; *P* = 0.178). Length of ICU stay and hospital stay was markedly reduced in patients undergoing CVP measurement within 3 hours (median 6.2 and 10.9 days, respectively, *P* < 0.001). The addition of the mean perfusion pressure initial, CVP, and the magnitude of the CVP change within 48 hours to the model significantly increased model discrimination (area under the receiver operating characteristic curve: 0.867 and 0.780, respectively, *P* < 0.001).

**Conclusions:**

These findings suggest that CVP measurement alone has little effect on the outcome of septic AKI. Nonetheless, initial CVP levels and the dynamic changes in CVP within the first 48 hours after ICU admission and the mean perfusion pressure initial can improve the accuracy of outcome prediction models.

## 1. Introduction

Sepsis, a systemic inflammatory response syndrome, is caused by an infection and is a common and serious complication in critically ill patients, especially in the intensive care unit (ICU) [1, 2]. Kidneys are among the organs most susceptible to sepsis-induced damage [3]. Renal hypoperfusion has been regarded as an important cause of septic AKI for a long time, and because septic AKI is considered reversible, certain authors have done some research and found that early monitoring intended to detect renal hypoperfusion as well as timely correction is helpful for improving the outcomes of the patients [4, 5]. Central venous pressure (CVP) measurements are often applied for assessing volume status and volume responsiveness at the bedside [6]. The key points of the “6-hour resuscitation bundle” of the Surviving Sepsis Campaign Guidelines recommend rapid infusion of intravenous fluids to achieve a CVP of >8 mmHg [4, 7]. Moreover, high CVP should not be ignored because elevated values have proved to be strongly associated with mortality [8]. Nevertheless, increasing evidence suggests that total renal blood flow (RBF) is not universally impaired during sepsis, and AKI may occur when there is no decrease or even when there is an increase in RBF [9, 10]. Based on sepsis-3 criteria and Medical Information Mart for Intensive Care IV (MIMIC IV) database, we aimed to elucidate the effect of baseline or early changes in CVP within the first 48 hours after ICU admission on short-term outcomes among patients with septic AKI. In addition, we built a mortality prediction model for septic AKI and compare it to the validation dataset from the emergency intensive care unit (eICU).

## 2. Materials and Methods

### 2.1. Data Sources

We conducted a retrospective cohort study based on the MIMIC-IV (version 1.0) database [11] (source of the training set), which contains comprehensive and high-quality data on over 40,000 patients admitted to ICUs at the Beth Israel Deaconess Medical Center. We completed the Collaborative Institutional Training Initiative examination (Certification number 43357010 for author Y.X.) to gain access to the database. We used a dataset from the eICU (version 2.0.1) collaborative research database for validation [12]. Ethics approval for database access was received from the Massachusetts Institute of Technology (Cambridge, MA, USA) and Beth Israel Deaconess Medical Center (Boston, MA, USA). Because the patient data in the databases are anonymous, signed informed consent of the subjects was not required for this study.

### 2.2. Selection of Participants

Inclusion criteria for the patients were (i) adult age (≥18 years), (ii) confirmed sepsis diagnosis based on the sepsis-3 criteria [1], (iii) diagnosis and staging of AKI performed according to the kidney disease improving global outcomes (KDGIO) criteria [13], and (iv) CVP measured within 24 hours after ICU admission. The exclusion criteria were as follows: (i) multiple admissions, (ii) ICU stay < 48 hours, (iii) missing important data (demographics or CVP data recorded during the first 48 hours), (iv) preexisting chronic kidney disease [14], and (v) severe heart insufficiency (New York Heart Association III-IV).

### 2.3. Study Variables

Baseline data included age, sex, ethnicity, ICU type, and disease severity at admission as measured by the sequential organ failure assessment (SOFA) score [15], acute physiology score III (APS-III) [16], and Charlson comorbidity index [17]. Hemodynamic variables including mean arterial pressure (MAP), heart rate, lactic acid (Lac), stroke volume (SV), and cardiac index (CI) were measured during the first 24 hours in the ICU. Data on inflammatory indicators (including white blood cell count (WBC) and neutrophil-to-lymphocyte ratio (NLR)) and renal function index (including blood urea nitrogen (BUN) and serum creatinine (SCr)) were collected from patients' records covering the first 24 hours after ICU admission. Medical interventions included the use of vasopressors, mechanical ventilation (MV), and renal replacement therapy (RRT) in the first 24 hours after ICU admission. The mean perfusion pressure (MPP) was calculated as MAP minus CVP [18]. We also calculated fluid load (the total fluids in–the total fluids out) by the patient weight in kilograms [19].

A baseline value was defined as the first measurement within 24 hours after ICU admission (designated as day 0 (D0)). Variables measured between hours 24 and 48 after ICU admission were attributed to day 1 (D1). For individuals with more than one record during any period, the earliest record was used. The change in CVP (*Δ*CVP) was defined as follows:

(1) $$\begin{array}{c} \Delta \text{CVP}=\frac{\text{CVPD}1\;\left(\text{at }24\text{ to }48\text{ h after admission}\right)-\text{CVPD}0\;\left(\text{in the first }24\text{ hours of admission}\right)}{\text{CVPD}0}. \end{array}$$

### 2.4. Outcome Variables

The primary outcome metric in the present study was 28-day mortality. Secondary outcome metrics included the length of ICU stay, length of hospital stay, and in-hospital mortality.

### 2.5. Statistical Analysis

Continuous variables were expressed as the mean ± standard deviation, and group comparisons were performed by the Kruskal-Wallis test (multiple classes of variables) and Mann–Whitney *U* test (dichotomous variables) for variables with nonnormal distributions and by the independent-sample *t*-test when the distribution was normal. Frequencies and proportions were estimated for categorical variables and were compared by the chi-squared test or Fisher's test.

We first subdivided the patients into two groups based on whether they required CVP monitoring, and baseline characteristics were compared between the two groups. To reduce the “differential deviation” originating from the whole sample and the influence of potential confounding factors, propensity score matching (PSM) was applied to compile better-matching groups. We then carried out multivariate regression analysis to clarify the relation between CVP measurement and 28-day mortality, whereas a univariate regression model after PSM was employed to validate our findings. Patients were grouped by hours depending on the earliest time that the first CVP measurement could be done (<3 h, 4-6 h, 7-12 h, and 13-24 h), and meanwhile, we show its corresponding hemodynamic indexes and explore the impact on outcomes. To reveal the effects of initial CVP levels on outcomes, we divided the matching groups into four subgroups based on the baseline CVP value: (i) no-CVP group, (ii) baseline CVP < 8 mmHg (1 mmHg = 0.133 kPa), (iii) baseline CVP ≥ 8 and ≤12 mmHg, and (iv) baseline CVP > 12 mmHg [7]. We also examined the relationship between *Δ*CVP and 28-day mortality. The patients were divided into three groups based on *Δ*CVP: (i) *Δ*CVP of ≤0 (reference group), (ii) *Δ*CVP of >0 to ≤1, and (iii) *Δ*CVP of >1.

Baseline variables that were considered clinically relevant or that showed a univariate relationship with the outcome have *P* < 0.05. To more comprehensively assess the effects of CVP measurements on the accuracy of outcome prediction, we compared several models: model A included the following baseline characteristics: age, sex, SOFA score, APS-III score, Charlson comorbidity index, use of mechanical ventilation, use of RRT, use of vasopressors, MAP, SV, Lac, BUN, SCr, and NLR; model B included MPP in addition to all the variables of model A; model C included initial CVP level in addition to all the variables of model A; model D included *Δ*CVP in addition to all the variables of model A; and model E included initial CVP level and *Δ*CVP in addition to all the variables of model A. A nomogram was then constructed based on a model with the best performance. This nomogram was tested on the validation set in terms of discrimination and calibration. Receiver operating characteristic (ROC) curve analysis was performed to determine the diagnostic value and clinical utility of the model. All statistical analyses were performed in RStudio 1.4.1106 (R version 4.1.0) [20]. Data were considered significant when the two-tailed *P* value was less than 0.05.

## 3. Results

### 3.1. Basic Characteristics

**Figure 1:**
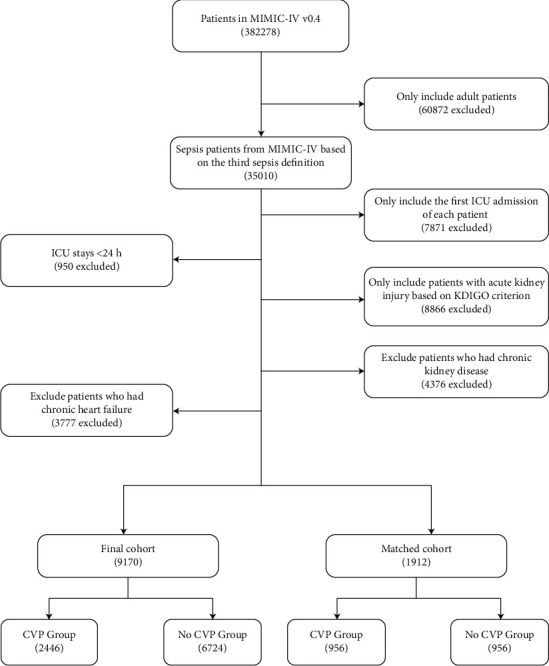
Flowchart of training set. MIMIC-IV = Medical Information Mart for Intensive Care IV database; ICU = intensive care unit; KDIGO = kidney disease improving global outcomes; CVP = central venous pressure.

A flowchart of patient selection is shown in Figure 1. Among the 9170 patients in the training set, there were 2446 patients in the CVP group and 6724 patients in the non-CVP group. Baseline data on all these patients are presented in Table 1. In the original cohort, there were 5458 (59.5%) males, and the median age was 63.0 years (range, 18-91 years). A total of 1971 patients (21.5%) had grade ≥ 2 AKI (1807 patients with grade 2 and 164 with grade 3). Patients in the CVP group had a higher mean SOFA score (7.5 vs. 6.6; *P* < 0.001). AKI severity, SCr, and BUN were statistically significantly higher in the CVP group than in the non-CVP group (all *P* < 0.001). Patients in the CVP group were also more likely to receive MV (69.9 vs. 40.7; *P* < 0.001), vasopressors (12.2 vs. 4.6; *P* < 0.001), and RRT (3.2 vs. 2.9; *P* < 0.001) within the first 24 hours after ICU admission. After PSM, all the variables were similar between the two groups.

### 3.2. Association between CVP Measurement and Clinical Outcomes

**Figure 2:**
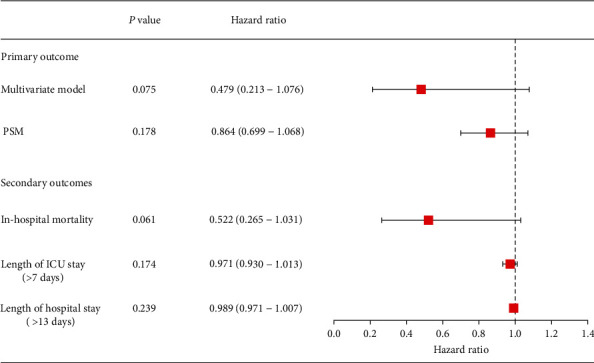
Association between CVP measurement and 28-day mortality. Analysis of the primary outcome metric via two models: (1) a multivariate logistic regression model (adjusted by age, sex, SOFA score, APS-III score, Charlson comorbidity index, use of mechanical ventilation, use of RRT, use of vasopressors, MAP, SV, Lac, BUN, SCr, and NLR) and (2) PSM model. PSM = propensity score matching; ICU = intensive care unit; SOFA = sequential organ failure assessment; APS-III = acute physiology score III; RRT = renal replacement therapy; MAP = mean arterial pressure; SV = stroke volume; Lac = lactic acid; NLR = neutrophil-to-lymphocyte ratio; BUN = blood urea nitrogen; SCr = serum creatinine.

Multivariate logistic regression analyses showed that CVP monitoring had no significant effect on 28-day mortality in patients with septic AKI (odds ratio = 0.479; 95% confidence interval, 0.213-1.076, *P* = 0.075). This was confirmed by adjustments (PSM; Figure 2). In patients with CVP measurement, early measurement (≤3 h) decreased ICU length of stay and hospital length of stay (6.2 and 10.9, respectively, *P* < 0.05); however, no obvious difference in in-hospital mortality and 28-day mortality was observed among groups (Table 2). A relatively higher CVP and lower MPP could be found in patients with early CVP measurement (17.3 and 90.0, respectively, *P* < 0.05).

### 3.3. Association of Changes in CVP Levels with Clinical Outcomes

**Figure 3:**
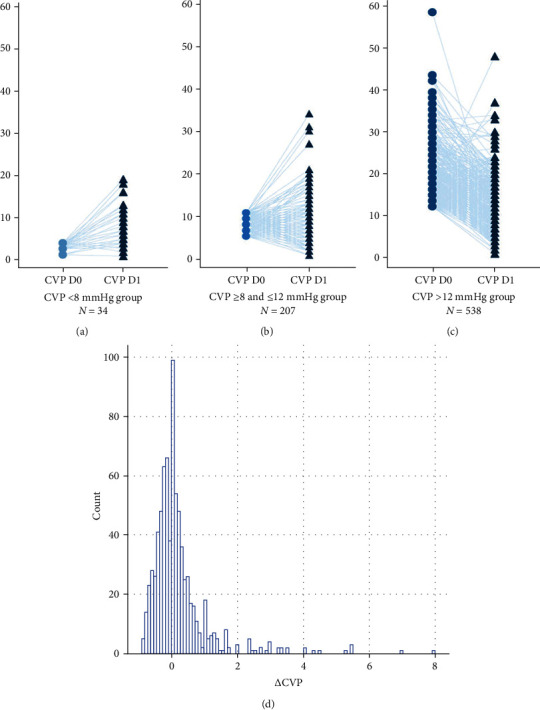
Dynamic changes in CVP during first 48 hours and the distribution of the 779 patients by *Δ*CVP values. (a) Dynamic changes in initial CVP < 8 mmHg group. (b) Dynamic changes in initial CVP ≥ 8 and ≤12 mmHg group. (c) Dynamic changes in initial CVP > 12 mmHg group. (d) The distribution of the 779 patients by *Δ*CVP values. Initial CVP level measure in the first 24 hours after admission. ΔCVP = [CVPD1 (at 24 to 48 h after admission) − CVPD0 (in the first 24 hours of admission)]/CVPD0.

Of the 1912 patients enrolled in the CVP group (PSM cohort), 779 had complete data at all time points and were included in the current report. In the CVP < 8 mmHg group, 29.4% satisfied the target CVP level (8–12 mmHg) and only one patient had a minor decrease in the CVP level (ΔCVP = −0.3) (Figure 3(a)). The mean value of CVP D1 for the CVP < 8 mmHg group was 8.4 ± 4.8 mmHg, for CVP ≥ 8 and ≤12 mmHg group 9.4 ± 5.2 mmHg, and for CVP > 12 mmHg group 12.8 ± 5.6 mmHg (Figures 3(a)–3(c)). Although CVP tended to decrease in the CVP > 12 mmHg group, we found a less pronounced decline in the CVP level (*Δ*CVP range, -0.9 to -0.1). General *Δ*CVP ranged from −0.9 to 8 (median, 0.2; interquartile range −0.3 to 0.3) (Figure 3(d)). Subgroup analysis is based on initial CVP level and *Δ*CVP (Table 3). The CVP > 12 mmHg group had significantly greater 28-day mortality rates (79.7%, *P* < 0.05) than the other groups (*vs*. CVP < 8 mmHg group and CVP ≥ 8 and ≤12 mmHg group; 2.4% and 17.9%, respectively). Similar results indicated significant associations between the high initial CVP level and hospital mortality. In the unadjusted logistic regression analysis, *Δ*CVP greater than 1 was associated with a decreased risk for 28-day mortality (OR = 0.388; 95%CI = 0.172-0.876; *P* = 0.023). Analyses of in-hospital mortality yielded similar findings (OR = 0.378; 95%CI = 0.167-0.853; *P* = 0.019).

### 3.4. Development of the Model for Mortality Prediction in Septic AKI and Model Performance

**Figure 4:**
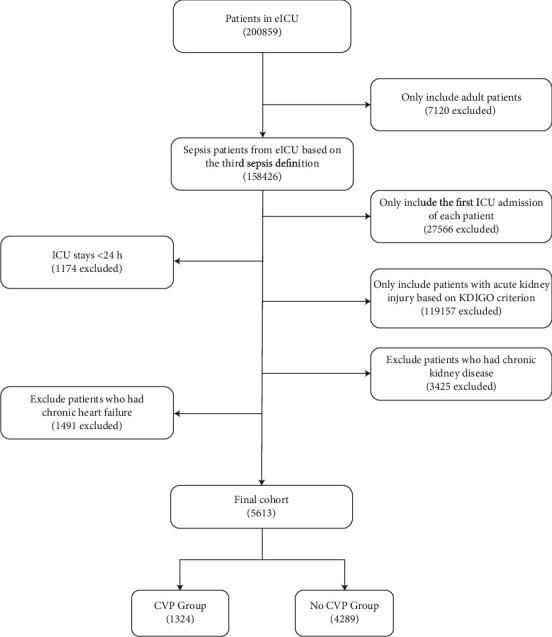
Flowchart of validation set. eICU = emergency intensive care unit; KDIGO = kidney disease improving global outcomes; ICU = intensive care unit; CVP = central venous pressure.

Multivariate logistic analysis of potential predictive variables was performed in the matched cohort (779 had complete data) and validation set. A flowchart of patient selection for the validation set is shown in Figure 4. The baseline characteristics for the validation set are summarized in Table 4.

**Figure 5:**
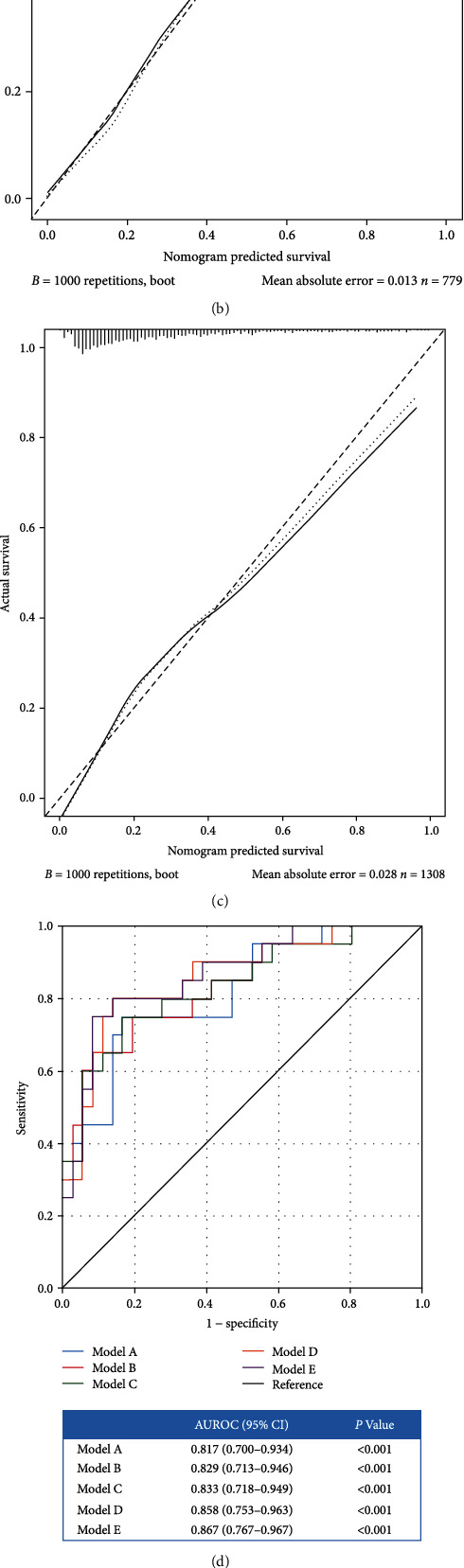
The construction of the structural models and the analysis of the results. (a) Nomogram plots based on the multiple logistic regression model are displayed by means of data collected from the cohorts after adjustment by PSM. The scores from each item were also summed to obtain an overall score, where greater probability of 28-day death might be detected. Calibration plots of the training set and validation set are displayed too. Calibration curves for the training set (b) and validation set (c). ROC curves of the four models for the training set (d) and validation set (e). Model A (blue line) included the following baseline characteristics: age, sex, SOFA score, APS-III score, Charlson comorbidity index, use of mechanical ventilation, use of RRT, use of vasopressors, MAP, SV, Lac, BUN, SCr, and NLR; model B (red line) included MPP in addition to all the variables of model A; model C (green line) included initial CVP level in addition to all the variables of model A; model D (orange line) included *Δ*CVP in addition to all the variables of model A; model E (purple line) included MPP, initial CVP level, and *Δ*CVP in addition to all the variables of model A. Initial CVP level measure in the first 24 hours after admission. ΔCVP = [CVPD1 (at 24 to 48 h after admission) − CVPD0 (in the first 24 hours of admission)]/CVPD0. SOFA = sequential organ failure assessment; APS-III = acute physiology score III; RRT = renal replacement therapy; SV = stroke volume; MAP = mean arterial pressure; BUN = blood urea nitrogen; SCr = serum creatinine; NLR = neutrophil-to-lymphocyte ratio; Lac = lactic acid; MPP = mean perfusion pressure; CVP = central venous pressure; ROC = receiver operator characteristic.

Based on this model, a nomogram was plotted to predict the probability of death of patients with septic AKI within 28 days (Figure 5(a)). Calibration curves were constructed for the training set and validation set (Figures 5(b) and 5(c)). In both sets, the apparent curve and bias-corrected curve slightly deviated from the reference line, but a good agreement between observation and prediction was observed. In ROC analysis, the addition of MPP or initial CVP or *Δ*CVP to model A significantly increased model discrimination in the training set (AUC: 0.829, 0.833, and 0.858, respectively, Figure 5(d)). Similar results were obtained in the external validation set (AUC: 0.759, 0.761, and 0.777, respectively, Figure 5(e)). Furthermore, model E showed good performance, with an area under the curve (AUC) of 0.867 in the training set and 0.780 in the external validation set.

## 4. Discussion

In our study, we found that CVP measurement had no effect on 28-day mortality among patients with septic AKI. There were also no statistically significant differences in ICU and total hospital length of stay and hospital mortality. The model that combines MPP, initial CVP, and *Δ*CVP indices best predicts 28-day mortality among patients with septic AKI (AUC: 0.867 in the training set and 0.780 in the external validation set), significantly outperforming the predictive ability of the base model (model A: AUC = 0.817 in the training set and 0.760 in the external validation set).

### 4.1. The Utility of CVP Monitoring

A properly measured CVP can serve as a guide to right ventricular filling pressure [21]. Othman et al. have found that maintaining a specific CVP level can help to keep patients hemodynamically stable, maintain renal perfusion, and prevent further ischemic injury due to ongoing renal dysfunction [22]. On the other hand, there is an inverse correlation between CVP and venous return, and an increase in CVP is suggestive of reduced venous return, which may disturb microcirculatory blood flow and cause organ failure [9]. A retrospective study on 2557 patients who have undergone right heart catheterization shows that increased CVP is associated with impaired renal function and independently correlates with all-cause mortality [23]. In other words, patients with either low or high CVP levels have higher death rates. Nevertheless, in clinical practice, we have found it difficult to determine the best level of CVP, and monitoring of CVP alone cannot accurately predict fluid responsiveness [22]. Therefore, it came as no surprise that the benefits of CVP monitoring in terms of clinical outcome were not found in our study. Moreover, our results are consistent with the findings of other studies, which indicate that patients in whom CVP is monitored have a higher prevalence of AKI than patients without this monitoring [24]. Nonetheless, this does not imply that we should completely give up on the measurement of CVP. Legrand et al. have reported that CVP is the only hemodynamic variable associated with the development of AKI; cardiac output, mixed venous oxygen saturation, and MAP do not predict AKI [25]. Although extreme CVP values were used in that study, CVP measurement was still useful in some respects. We also explored the association between the observational time points of CVP measurement and outcomes which shows early CVP measurement is associated with decreased ICU length of stay and hospital length of stay (6.2 and 10.9, respectively, *P* < 0.05). This view is supported by previous studies showing that the 28-day mortality in the early CVP measurement group was significantly lower than that in the control group (34.2% vs. 40.7%, *P* < 0.01) [26]. Some patients in our study had their first CVP measurement within 13 to 24 hours of admission, and their CVP levels, fluid load, and MPP were higher than those of other groups (median 19.0 mmHg, 4.8 mL/kg, and 93 mmHg, respectively). We speculate that this phenomenon may be related to fluid resuscitation; however, there was no advantage with respect to outcomes.

### 4.2. The Initial Values and Rates of Change of CVP

In our study, we found that high CVP levels at baseline were associated with higher 28-day and in-hospital mortality. The previous studies reported that initial high CVP was associated with a poor prognosis [27, 28]. Combined with information from literature review, we consider that high CVP group patients did not have adequate venous return because of excess fluid therapy or high intrathoracic pressure [28]. Many authors have stated that maintaining CVP < 8 mmHg during the early phase of septic shock can prevent further impairment of renal function [25, 29]. However, it is not clear how low the CVP needs to be in patients with sepsis-associated AKI. Further study showed that a 1 mmHg increase in the lowest CVP value during the first 72 hours after ICU admission increased the odds of all-cause mortality during a 90-day period by 3.1% [27]. This is seemingly in opposition to our results. Our study reveals that elevated CVP portends a better prognosis, which is independently associated with a decrease in 28-day mortality. The contradictory results in the study may be attributed to the following reasons. First, patients with an elevated CVP (particularly if ΔCVP > 1) were more likely to have a lower initial CVP level. Additionally, CVP levels are still within 8-12 mmHg after CVP elevation. The results highlight the need for personalized dosimetry.

### 4.3. Models Predicting 28-Day Mortality

In this work, final models were constructed considering available baseline data and MPP, initial CVP levels, and *Δ*CVP; these models manifested high diagnostic accuracy. In the present study, as in most other studies, the models included such variables as sex [30], severity of illness [31, 32], therapeutic interventions [32], and laboratory tests [33]. In this study, NLR was used to evaluate the infection status. NLR is an innovative inflammatory biomarker derived from combined neutrophil and lymphocyte counts [34]. NLR is dual stimulation of two innate immune pathways: nonspecific inflammation triggers neutrophils, whereas a decreased lymphocyte count suggests that the body is under stress or has poor immunity [35]. Recently, several studies have shown that an elevated NLR was associated with poor prognosis in critical patients; this appears to be consistent with the observation in our study [36]. In this study, we used MPP as an indicator which reflects changes in intrarenal perfusion, and the result shows there was an increased risk of 28-day death with decreasing MPP levels, and meanwhile, including MPP in the model could improve the predictive ability of the base model. Some studies indicated that lower MPP was strongly associated with the development of AKI, and furthermore, Peng et al. indicated that increased MPP-CV in the first 24 h after ICU admission was associated with deterioration of renal function in subsequent 48 h [18, 37, 38]. In the present study, MPP, CVP, *Δ*CVP, and baseline data were used in the final model because they are easier and safer to measure and more readily available. We also tested the model for external validity; this analysis confirmed the validity of our model and its generalizability to external cohorts.

## 5. Limitations

This study has several limitations. First, the use of PSM imposes restrictions on sample size for such a study and potentially limits the ability to precisely estimate the effects. Meanwhile, the results of our study should be interpreted with caution because residual confounding factors cannot be ruled out completely. Second, this is a retrospective cohort study based on electronic medical records, whereas the reliability of CVP measurements has been questioned, and CVP is known to be influenced by many factors, such as errors related to the positioning of the zero level or reading error [6]. Lastly, because the study is based on an observational database, the results reported in our study should be regarded only as a reference and must be further verified. Additional high-quality randomized trials with larger sample sizes are needed to develop risk models optimized for patients with septic AKI.

## 6. Conclusion

CVP measurement alone has little effect on the outcome of patients with septic AKI. Nevertheless, as a hemodynamic indicator, when combined with AKI risk factors, MPP, initial CVP levels, and the dynamic changes in CVP within the first 48 hours after ICU admission can improve the accuracy of outcome prediction models.

## Figures and Tables

**Table 1:** Comparisons of baseline characteristics between the original cohort and matched cohort.

Variables	Original cohort			Matched cohort		
	No CVP group *N* = 6724	CVP group *N* = 2446	SMD	No CVP group *N* = 956	CVP group *N* = 956	SMD
Age (year, mean (SD))	61.2 (16.7)	63.2 (14.7)	0.123	59.8 (16.2)	60.7 (16.2)	0.057
Male (%)	3888 (57.8)	1570 (64.2)	0.131	578 (60.5)	573 (59.9)	0.011
Ethnicity, *n* (%)						
American Indian or Alaska Native	10 (0.1)	1 (0.0)	0.200	6 (0.6)	0 (0.0)	0.225
Asian	181 (2.7)	55 (2.2)		25 (2.6)	31 (3.2)	
Black/African American	475 (7.1)	98 (4.0)		52 (5.4)	52 (5.4)	
Hispanic/Latino	232 (3.5)	51 (2.1)		38 (4.0)	20 (2.1)	
White	4307 (64.1)	1744 (71.3)		552 (57.7)	625 (65.4)	
Other	325 (4.8)	134 (5.5)		49 (5.1)	48 (5.0)	
Unknown	1194 (17.8)	363 (14.8)		234 (24.5)	180 (18.8)	
First care unit, *n* (%)						
CVICU	796 (11.8)	1392 (56.9)	1.122	242 (25.3)	266 (27.8)	0.256
CCU	285 (4.2)	83 (3.4)		53 (5.5)	61 (6.4)	
MICU	1479 (22.0)	327 (13.4)		284 (29.7)	243 (25.4)	
MICU/SICU	1215 (18.1)	213 (8.7)		124 (13.0)	144 (15.1)	
Neuro intermediate	67 (1.0)	0 (0.0)		3 (0.3)	0 (0.0)	
Neuro SICU	303 (4.5)	5 (0.2)		30 (3.1)	5 (0.5)	
SICU	1300 (19.3)	233 (9.5)		103 (10.8)	130 (13.6)	
TSICU	1279 (19.0)	193 (7.9)		117 (12.2)	107 (11.2)	
Severity of illness (mean (SD))						
SOFA score	6.6 (3.8)	7.5 (4.1)	0.226	8.7 (4.6)	9.0 (4.4)	0.063
APS-III score	46.3 (11.5)	43.4 (13.0)	0.240	47.6 (11.5)	48.4 (11.2)	0.064
Charlson comorbidity index	4.9 (2.7)	4.5 (2.1)	0.173	4.6 (2.7)	4.5 (2.4)	0.057
Hemodynamic variables measured in the first 24 hours of admission to ICU (mean (SD))						
MAP (mmHg)	107.8 (26.6)	104.8 (30.6)	0.107	107.0 (30.9)	108.3 (34.9)	0.038
HR (bpm)	108.8 (21.1)	105.1 (20.2)	0.179	109.9 (22.1)	111.1 (22.8)	0.052
CI (L/min/m^2^)	3.2 (0.9)	3.1 (0.9)	0.181	3.2 (0.9)	3.1 (0.9)	0.094
SV (mL)	63.6 (23.3)	65.6 (26.8)	0.083	58.1 (25.1)	63.4 (27.6)	0.201
Lac (mmol/L)	3.2 (3.3)	3.6 (2.7)	0.129	3.9 (3.9)	3.9 (3.2)	0.009
Inflammatory indicators measured in the first 24 hours of admission to ICU (mean (SD))						
WBC (×10^9^/L)	15.9 (12.9)	16.9 (9.1)	0.090	17.5 (18.0)	17.9 (11.3)	0.023
NLR	3.9 (2.7)	3.6 (2.4)	0.087	3.8 (1.2)	3.5 (1.4)	0.029
Renal function index measured in the first 24 hours of admission to ICU (mean (SD))						
BUN (mg/dL)	25.2 (20.4)	23.8 (16.9)	0.072	29.0 (26.2)	28.9 (20.7)	0.002
SCr (mg/dL)	1.3 (1.2)	1.3 (1.0)	0.008	1.6 (1.5)	1.6 (1.4)	0.030
AKI stage, *n* (%)						
I	5113 (76.0)	2086 (85.3)	0.238	771 (80.6)	757 (79.2)	0.066
II	1470 (21.9)	337 (13.8)		169 (17.7)	188 (19.7)	
III	141 (2.1)	23 (0.9)		16 (1.7)	11 (1.2)	
Interventions in the first 24 hours of admission to ICU, *n* (%)						
MV use	2734 (40.7)	1709 (69.9)	0.615	657 (68.7)	613 (64.1)	0.098
Vasopressor use	306 (4.6)	298 (12.2)	0.278	155 (16.2)	185 (19.4)	0.082
RRT use	196 (2.9)	78 (3.2)	0.016	59 (6.2)	58 (6.1)	0.004

CVP = central venous pressure; SMD = standard mean difference; CVICU = cardiovascular intensive care unit; CCU = coronary care unit; MICU = medical intensive care; SICU = surgical intensive care unit; TSICU = trauma surgical intensive care unit; SOFA = sequential organ failure assessment; APS-III = acute physiology score III; MAP = mean arterial pressure; HR = heart rate; CI = cardiac index; SV = stroke volume; Lac = lactic acid; WBC = white blood cell; NLR = neutrophil-to-lymphocyte ratio; BUN = blood urea nitrogen; SCr = serum creatinine; AKI = acute kidney injury; MV = mechanical ventilation; RRT = renal replacement therapy.

**Table 2:** Comparison of the effects of different CVP administration times on outcomes in propensity score-matched cohorts (*N* = 1912).

Variables	≤3 h *N* = 449	4~6 h *N* = 231	7~12 h *N* = 183	13~24 h *N* = 93	*P* value
CVP (mmHg, mean (SD))	17.3 (7.4)	14.1 (7.4)^a^	12.8 (6.7)^a^	19.0 (7.5)^bc^	<0.001
MAP (mmHg, mean (SD))	107.3 (32.9)	107.0 (34.7)	110.3 (38.4)	112.1 (37.9)	0.259
CI (L/min/m^2^, mean (SD))	3.4 (1.0)	2.7 (0.7)	3.0 (0.8)	3.1 (1.6)	0.181
Lac (mmol/L, mean (SD))	3.9 (3.5)	3.7 (2.8)	4.1 (3.2)	3.9 (2.6)	0.161
Fluid load (mL/kg, mean (SD))	4.5 (1.7)	4.4 (1.5)	4.7 (1.9)	4.8 (1.4)	0.095
MPP (mmHg, mean (SD))	90.0 (33.2)	92.9 (36.0)	97.5 (38.5)^a^	93.0 (37.5)	0.002
Primary outcome					
28-day mortality, *n* (%)	96 (21.4)	53 (22.9)	41 (22.4)	22 (23.7)	0.947
Secondary outcomes					
In-hospital mortality, *n* (%)	99 (22.0)	53 (22.9)	42 (23.0)	24 (25.8)	0.956
Length of ICU stay (day, mean (SD))	6.2 (8.6)	7.1 (8.0)^a^	7.1 (6.8)	6.9 (6.2)	0.021
Length of hospital stay (day, mean (SD))	10.9 (8.0)	12.2 (10.2)	11.3 (12.3)	12.8 (11.7)^abc^	<0.001

CVP level measure in the first 24 hours of admission to ICU. ^a^*P* < 0.05 vs. CVP measurement within 3 hours; ^b^*P* < 0.05 vs. CVP measurement from 4 to 6 hours; ^c^*P* < 0.05 vs. CVP measurement from 7 to 12 hours; a two-tailed *P* value of <0.05 was considered statistically significant. CVP = central venous pressure; MAP = mean arterial pressure; CI = cardiac index; Lac = lactic acid; MPP = the mean perfusion pressure; ICU = intensive care unit.

**Table 3:** Outcome difference in initial level and rate of change in CVP levels in propensity score-matched cohorts.

Variables	28-day mortality		In-hospital mortality		Length of ICU stay (>7 days)		Length of hospital stay (>13 days)	
	OR (95% CI)	*P* value	OR (95% CI)	*P* value	Coefficient (95% CI)	*P* value	Coefficient (95% CI)	*P* value
Initial CVP (*n* = 956)								
<8 mmHg (*n* = 41)	1.000 (reference)		1.000 (reference)		1.000 (reference)		1.000 (reference)	
≥8, ≤12 mmHg (*n* = 258)	2.577 (1.142-5.818)	0.023	2.646 (1.173-5.972)	0.019	0.712 (0.419-1.210)	0.210	0.669 (0.395-1.134)	0.136
>12 mmHg (*n* = 657)	3.097 (1.357-7.070)	0.007	3.268 (1.433-7.452)	0.005	0.980 (0.569-1.689)	0.942	0.935 (0.544-1.607)	0.808
*Δ*CVP (*n* = 779)								
≤0 (*n* = 425)	1.000 (reference)		1.000 (reference)		1.000 (reference)		1.000 (reference)	
>0, ≤1 (*n* = 286)	1.202 (0.849-1.701)	0.300	1.235 (0.875-1.742)	0.229	0.791 (-0.191-1.773)	0.114	0.044 (-1.596-1.684)	0.958
>1 (*n* = 68)	0.388 (0.172-0.876)	0.023	0.378 (0.167-0.853)	0.019	1.050 (-0.841-2.941)	0.276	0.880 (-2.278-4.037)	0.585

ΔCVP = [CVPD1 (at 24 to 48 h after admission) − CVPD0 (in the first 24 hours of admission)]/CVPD0. A two-tailed *P* value of <0.05 was considered statistically significant. CVP = central venous pressure; ICU = intensive care unit; CI = confidence interval.

**Table 4:** Basic characteristics of the validation set (*N* = 5613).

Variables	No CVP group *N* = 4289	CVP group *N* = 1324	SMD
Age (year, mean (SD))	64.0 (16.1)	64.4 (14.2)	0.024
Male (%)	2030 (47.3)	716 (54.1)	0.135
Ethnicity, *n* (%)			
African American	615 (14.3)	141 (10.6)	0.129
Asian	77 (1.8)	30 (2.3)	
Caucasian	3153 (73.5)	1004 (75.8)	
Hispanic	159 (3.7)	45 (3.4)	
Native American	31 (0.7)	7 (0.5)	
Other/unknown	254 (5.9)	97 (7.3)	
First care unit, *n* (%)			
Cardiac ICU	278 (6.5)	65 (4.9)	0.414
CCU-CTICU	292 (6.8)	130 (9.8)	
CSICU	84 (2.0)	19 (1.4)	
CTICU	102 (2.4)	138 (10.4)	
Med-Surg ICU	2288 (53.3)	632 (47.7)	
MICU	614 (14.3)	162 (12.2)	
Neuro ICU	410 (9.6)	71 (5.4)	
SICU	221 (5.2)	107 (8.1)	
Severity of illness (mean (SD))			
SOFA score	7.3 (2.9)	9.6 (3.2)	0.772
APS-III score	62.6 (27.8)	81.9 (32.9)	0.635
Charlson comorbidity index	2.7 (1.4)	2.8 (1.3)	0.070
Hemodynamic variables measured in the first 24 hours of admission to ICU (mean (SD))			
MAP (mmHg)	67.3 (30.3)	64.6 (28.8)	0.093
HR (bpm)	85.4 (18.3)	89.3 (18.7)	0.213
CI (L/min/m^2^)	2.6 (0.7)	2.6 (0.8)	4.429
SV (mL)	62.0 (29.6)	63.1 (21.6)	0.098
Lac (mmol/L)	2.3 (2.5)	3.3 (3.6)	0.328
Inflammatory indicators measured in the first 24 hours of admission to ICU (mean (SD))			
WBC (×10^9^/L)	11.4 (8.4)	14.3 (10.8)	0.296
NLR	10.6 (15.6)	13.5 (16.0)	0.181
Renal function index measured in the first 24 hours of admission to ICU			
BUN (mg/dL)	30.8 (24.9)	37.2 (25.7)	0.255
SCr (mg/dL)	2.0 (2.1)	2.3 (2.0)	0.141
AKI stage			
I	3833 (89.6)	1172 (88.7)	0.027
II	231 (5.4)	77 (5.8)	
III	216 (5.0)	72 (5.5)	
Interventions in the first 24 hours of admission to ICU, *n* (%)			
MV use	1176 (27.4)	785 (59.3)	0.679
Vasopressor use	16 (0.4)	15 (1.1)	0.088
RRT use	11 (0.3)	9 (0.7)	0.062

CVP = central venous pressure; SMD = standard mean difference; CVICU = cardiovascular intensive care unit; CCU = coronary care unit; MICU = medical intensive care; SICU = surgical intensive care unit; TSICU = trauma surgical intensive care unit; SOFA = sequential organ failure assessment; APS-III = acute physiology score III; MAP = mean arterial pressure; HR = heart rate; CI = cardiac index; SV = stroke volume; Lac = lactic acid; WBC = white blood cell; NLR = neutrophil-to-lymphocyte ratio; BUN = blood urea nitrogen; SCr = serum creatinine; AKI = acute kidney injury; MV = mechanical ventilation; RRT = renal replacement therapy.

## Data Availability

The data that support the findings of this study are available from the corresponding author upon reasonable request.

## Abbreviations

ICU: Intensive care unit
AKI: Acute kidney injury
CVP: Central venous pressure
RBF: Renal blood flow
MIMIC-IV: Medical Information Mart for Intensive Care IV
eICU: Emergency intensive care unit
KDGIO: Kidney disease improving global outcomes
SOFA: Sequential organ failure assessment
APS-III: Acute physiology score III
MAP: Mean arterial pressure
WBC: White blood cell
NLR: Neutrophil-to-lymphocyte ratio
CRP: C-reactive protein
BUN: Blood urea nitrogen
SCr: Serum creatinine
Lac: Lactic acid
MV: Mechanical ventilation
RRT: Renal replacement therapy
PSM: Propensity score matching
ROC: Receiver operator characteristic
DCA: Decision curve analysis
AUC: Area under the curve
MPP: The mean perfusion pressure.
